# Extracorporeal membrane oxygenation for refractory arrhythmia in a neonate carrying an SCN5A variant: a case report

**DOI:** 10.1186/s43044-026-00791-y

**Published:** 2026-10-30

**Authors:** Chen Yixian, Gan Yuan, Zhang Senxiong, Lei Xianqiang, Jiang Yongjiang, Tang Mingyun, Liu Yunzhi, Lv Yanxing, Wei Yijun

**Affiliations:** Liuzhou Hospital of Guangzhou Women and Children’s Medical Center, Liuzhou, China

**Keywords:** Neonate, Malignant arrhythmia, SCN5A gene, Extracorporeal membrane oxygenation

## Abstract

**Background:**

Neonatal malignant arrhythmias refractory to conventional antiarrhythmic therapy are life-threatening and pose a major therapeutic challenge, particularly in premature infants.

**Case presentation:**

We report a premature infant born at 36 + 2 weeks of gestation who developed ventricular tachycardia and cardiac arrest following resuscitation for perinatal asphyxia. As lidocaine and amiodarone failed to control the rhythm, veno‑arterial ECMO was initiated shortly thereafter. After ECMO support, the rhythm converted to sinus and cardiac function improved, allowing successful weaning. Electrocardiography subsequently revealed a prolonged QT interval, which responded to oral mexiletine. Whole‑exome sequencing identified a heterozygous SCN5A c.1960G > A (p.Glu654Lys) variant inherited from the father, classified as a variant of uncertain significance (VUS).

**Conclusions:**

This case suggests that this specific VUS may be a candidate pathogenic variant, and that early ECMO can be an effective salvage therapy when conventional drugs fail. Additionally, when genetic testing yields a VUS, the clinical phenotype and treatment response appear valuable for assessing pathogenicity in neonatal cases.

## Background

Arrhythmias are among the common cardiovascular conditions in the neonatal period. The cardiac conduction system in newborns is not yet fully developed, and its maturation process and functional changes constitute the anatomical and physiological basis for neonatal arrhythmias. In addition, various factors, including infection, hypoxia, asphyxia, electrolyte disturbances, medications, surgical interventions, and maternal autoimmune diseases during pregnancy, can cause myocardial injury in neonates and trigger different types of arrhythmias [1–3]. Neonatal asphyxia can lead to systemic hypoxia and hemodynamic alterations, resulting in multi-organ damage involving the nervous system, myocardium, liver, and kidneys, and may even cause disability or death [4]. However, in some neonates, arrhythmias cannot be readily explained by these acquired factors, suggesting that genetic predisposition may be the underlying cause of malignant arrhythmias. Here we report a premature infant who developed malignant arrhythmia, cardiac arrest, and heart failure after resuscitation for perinatal asphyxia. Conventional antiarrhythmic drugs were ineffective, and the infant was successfully managed with extracorporeal membrane oxygenation (ECMO). Whole-exome sequencing (WES) revealed a variant of uncertain significance in the SCN5A gene. We present the diagnostic and therapeutic course of this case.

## Case presentation

The patient was a premature infant born at 36 weeks and 2 days of gestation at an outside hospital via cesarean section due to fetal distress. Routine prenatal examinations had been unremarkable; however, fetal tachycardia was detected shortly before delivery. Apgar scores were 6 at 1 min and 7 at both 5 and 10 min. After resuscitation for perinatal asphyxia, the infant developed ventricular arrhythmia and cardiac arrest. Lidocaine administered at the outside hospital failed to restore sinus rhythm.

**Fig. 1:**
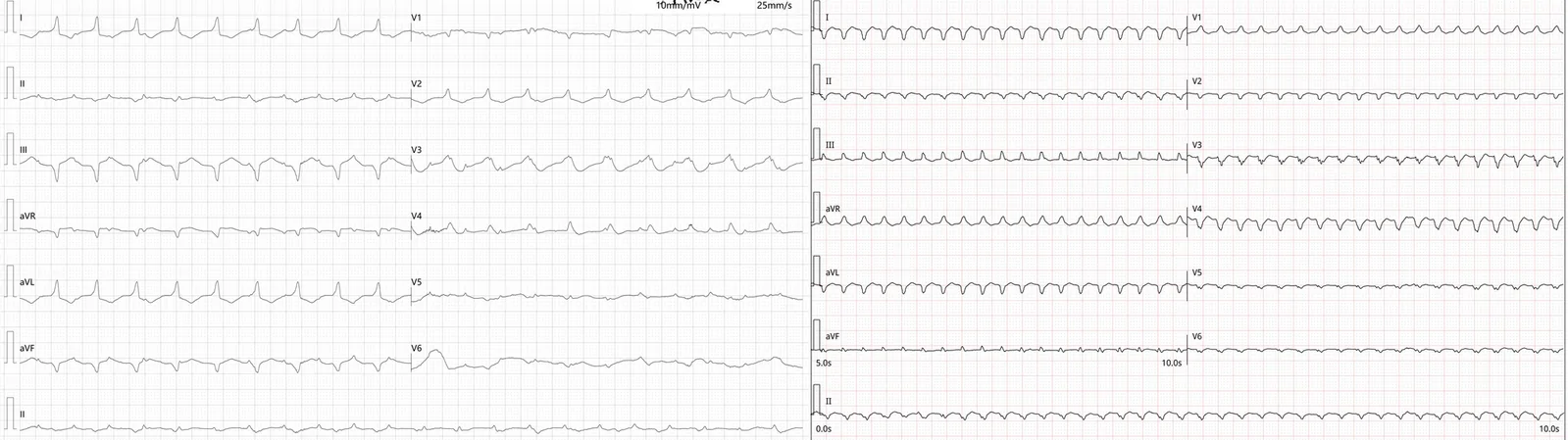
Electrocardiograms at two time points. On admission, the electrocardiogram showed sinus rhythm (atrial rate 136 bpm, ventricular rate 115 bpm), third-degree atrioventricular block, and an accelerated idioventricular rhythm, with a QTc of 536 ms (left). Approximately 10 h later, a monomorphic low-amplitude broad complex tachycardia in all leads. Although there is no clear ventriculo-atrial dissociation, the appearance is consistent with ventricular tachycardia (right)

Upon admission to our hospital, arterial blood gas analysis revealed: pH 7.178, PaO₂ 55.9 mmHg, PaCO₂ 19 mmHg, BE−21.5 mmol/L, and lactate 26.3 mmol/L. Cardiac biomarkers were markedly elevated: CK 1599 IU/L, CK-MB 530 IU/L, myoglobin > 1200 ng/mL, troponin I 25369.71 pg/mL, and B-type natriuretic peptide 20681.4 ng/L. Liver and renal function tests as well as electrolytes showed no significant abnormalities. Initial electrocardiography showed sinus rhythm with third-degree atrioventricular block and an accelerated idioventricular rhythm. Approximately 10 h later, the rhythm deteriorated to ventricular tachycardia with low voltage in all leads (Fig. 1). Electrocardiography demonstrated ventricular tachycardia with low voltage in all leads (Fig. 1). Echocardiography revealed an atrial septal defect (7.2 mm), patent ductus arteriosus (3.4 mm), pulmonary hypertension, and mild right ventricular enlargement. Cranial ultrasonography detected slightly hyperechoic foci in the bilateral subependymal regions, suggestive of grade I intraventricular hemorrhage.

**Fig. 2:**
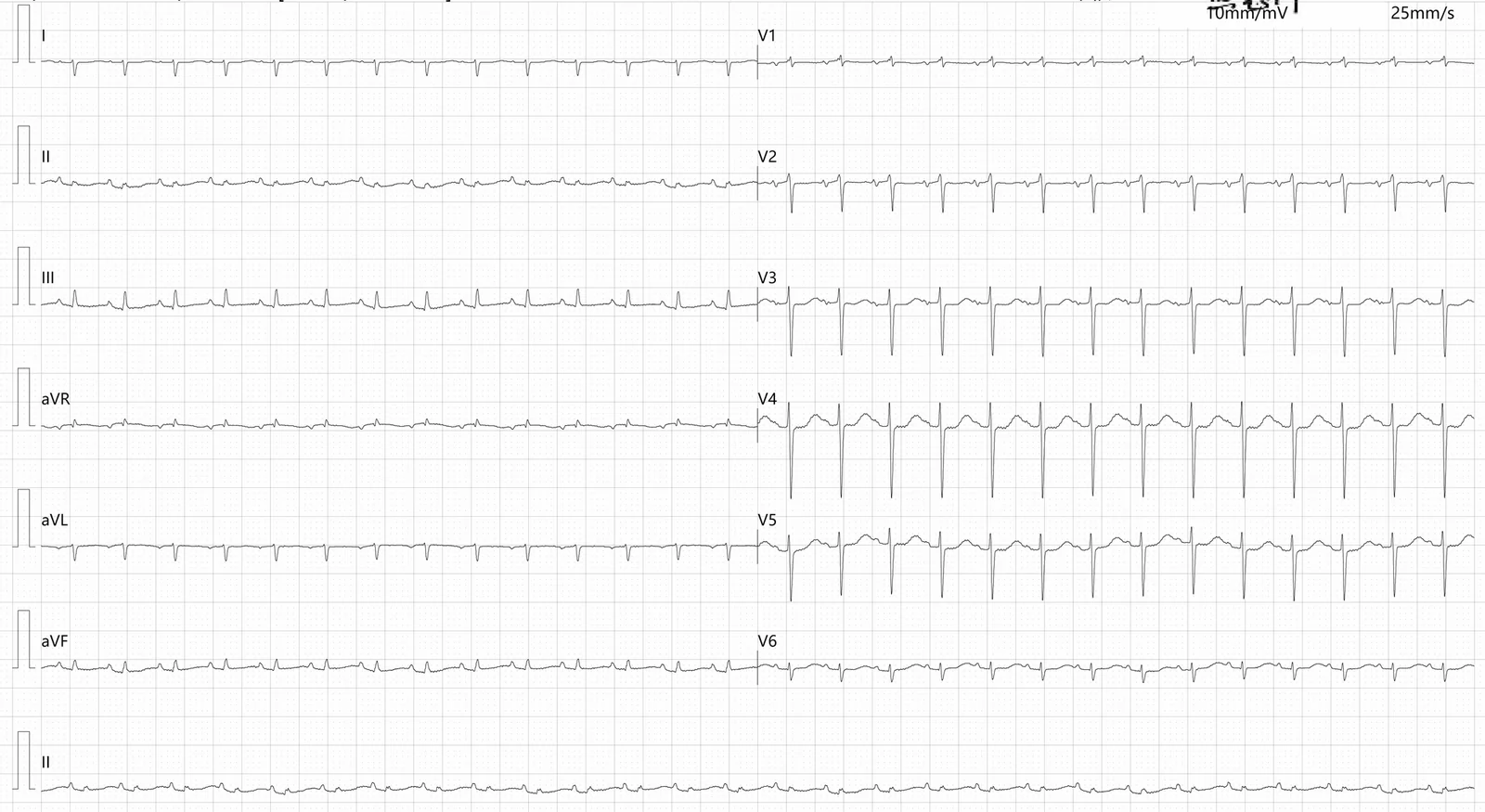
Electrocardiogram (lead II) on day 5 of admission, during ECMO support, showing sinus tachycardia, low voltage in the limb leads, clockwise rotation, and a prolonged QTc interval

Invasive mechanical ventilation was initiated, and dobutamine, dopamine, epinephrine, and hydrocortisone were administered for inotropic support and blood pressure stabilization. Amiodarone was given for arrhythmia control, and peritoneal dialysis was instituted. Despite these measures, the infant continued to have sustained arrhythmia, rising lactate levels, and worsening heart failure. ECMO was initiated at approximately 31 h of life, during which amiodarone was continued. At 12 h of ECMO support, the left ventricular ejection fraction was 21.5%. After ECMO initiation, the ventricular rhythm converted to sinus rhythm, and cardiac function recovered progressively. A prolonged QT interval was subsequently observed, and amiodarone was discontinued. ECMO was weaned after 86 h of support, with ligation of the cervical vessels. Invasive ventilation was continued, and the infant was extubated 5 days after ECMO decannulation, transitioning through non-invasive ventilation to nasal cannula oxygen. Four days after ECMO weaning, holter monitoring revealed sinus rhythm with short runs and paroxysmal atrial tachycardia, as well as occasional multifocal premature ventricular contractions. Propafenone and propranolol were started, but paroxysmal atrial and ventricular tachycardia still occurred intermittently. During this period, the peak QTc interval on electrocardiography reached 496 ms (Fig. 2). Based on whole-exome sequencing results, oral propranolol combined with mexiletine was initiated at 1.5 months of age, after which no further arrhythmias were documented.

Blood and urine screening for inherited metabolic disorders revealed no abnormalities. Whole-exome sequencing identified a heterozygous variant in the SCN5A gene on chromosome 3 (NM_000335.5:c.1960G > A, p.Glu654Lys), inherited from the father. The father’s ECG was normal, with a QTc of 387 ms, a PR interval of 153 ms, and a QRS duration of 94 ms. There was no family history of sudden death, syncope, or inherited arrhythmia syndrome. According to ACMG guidelines, this variant is classified as a variant of uncertain significance. Diseases associated with this gene include long QT syndrome type 3, dilated cardiomyopathy type 1E, progressive cardiac conduction defect, sudden infant death syndrome (susceptibility), sick sinus syndrome type 1, familial ventricular fibrillation type 1, familial atrial fibrillation type 10, progressive familial heart block type 1 A, and Brugada syndrome type 1.

At follow-up to 8 months of age, the infant demonstrated normal physical growth, was able to roll over, and could sit with support. Holter monitoring showed a normal QTc interval. Cranial MRI was normal, and echocardiography showed no abnormalities.

## Discussion

The most common sustained tachyarrhythmias in neonates are atrioventricular reentrant tachycardias, which may manifest with an overt accessory pathway or a concealed accessory pathway. Because the neonatal myocardium is immature, abnormally low or high ventricular rates can both lead to heart failure [5]. We describe a premature neonate with refractory ventricular tachycardia and cardiac arrest following perinatal asphyxia, in whom ECMO provided successful circulatory support and genetic testing later revealed an SCN5A variant of uncertain significance consistent with a long QT syndrome type 3 phenotype.

Neonatal ECMO is a life-saving technique used to treat critically ill neonates with severe cardiac and/or respiratory failure due to potentially reversible conditions that are unresponsive to maximal conventional therapy [6]. The application of this technology in neonates began in 1975 with the successful rescue of an infant with persistent pulmonary hypertension of the newborn (PPHN) [7]. Subsequently, neonatal respiratory failure caused by various conditions such as respiratory distress syndrome, meconium aspiration syndrome, and PPHN became the primary indications for ECMO. Over the past decade, the number of neonatal respiratory ECMO cases has declined, whereas cardiac indications have remained stable. Hospital survival for neonatal cardiac ECMO is approximately 36–55%, depending largely on the underlying indication. Preoperative and postoperative stabilization of congenital heart disease is the most common cardiac ECMO indication, followed by cardiac arrest, cardiogenic shock, cardiomyopathy and myocarditis, and refractory arrhythmias [5]. The present case involved a premature infant with cardiogenic shock secondary to refractory malignant arrhythmia, who continued to have sustained arrhythmia, hyperlactatemia, and heart failure despite treatment with multiple antiarrhythmic agents (lidocaine, amiodarone), invasive mechanical ventilation, vasoactive medications, and peritoneal dialysis. ECMO was initiated at 31 h of life, after which the rhythm converted to sinus, lactate levels normalized, cardiac function recovered, and the infant was successfully weaned. This case suggests that ECMO can be an effective therapeutic option for neonates with sustained malignant arrhythmia refractory to conventional medical therapy and complicated by heart failure, providing time for myocardial and electrophysiological recovery.

In the present case, potential etiologies of the arrhythmia were investigated from multiple perspectives, including maternal history during pregnancy, cardiac structural evaluation, and screening for inherited metabolic disorders. Ultimately, family-based WES identified a heterozygous SCN5A c.1960G > A (p.Glu654Lys) variant inherited from the father, which was classified as VUS according to ACMG guidelines. The SCN5A gene, located on chromosome 3, encodes NaV1.5, a voltage-gated sodium channel belonging to the sodium channel family subfamily 1 member 5. The influx of Na⁺from the extracellular environment into cardiomyocytes through the cardiac sodium channel generates the inward sodium current (INa), which underlies the generation and propagation of action potentials in working myocardial cells (atrial and ventricular myocytes) and specialized conduction tissue cells (such as Purkinje cells) [8].

Variants in the SCN5A gene result in dysfunction of the cardiac sodium channel [9]. The associated phenotypes are diverse and exhibit incomplete penetrance; affected individuals may be asymptomatic carriers or may present with life‑threatening arrhythmias, including Brugada syndrome, sick sinus syndrome, familial atrial fibrillation, and long QT syndrome type 3 (LQT3), among others [10–12]. The SCN5A c.1960G > A variant identified in this case has been previously reported in association with long QT syndrome [13]. QTc interval prolongation was observed in the early period after ECMO weaning. Several factors may have contributed to this finding, including ventricular unloading during ECMO support, the use of amiodarone, and inotropic support during the stabilization phase. However, the QTc remained prolonged after discontinuation of amiodarone and inotropic agents, suggesting that the QTc prolongation was not solely attributable to these reversible factors and may reflect an underlying repolarization abnormality related to the SCN5A variant. Therefore, the underlying cause of the arrhythmia in this case is most likely an inherited SCN5A-related overlap syndrome, in which LQT3 is the predominant phenotype and cardiac conduction disease is also present. Prematurity and perinatal asphyxia served as potential triggers. Given the presence of the SCN5A variant, the child requires lifelong cardiac care and repeated cardiac examinations. This missense variant is associated with an overlap syndrome that can manifest as long QT syndrome, Brugada syndrome, and/or cardiac conduction disease of varying severity. Long-term follow-up should include serial electrocardiography, echocardiography, and clinical assessment for arrhythmic events.

## Conclusions

This case suggests that the SCN5A c.1960G > A (p.Glu654Lys) variant of uncertain significance is highly suspicious as the genetic etiology underlying the malignant arrhythmia in this neonate, with a clinical phenotype consistent with an SCN5A-related overlap syndrome in which long QT syndrome type 3 is the core phenotype and a transient cardiac conduction abnormality was also documented. Prematurity and perinatal asphyxia may have served as triggering factors. For sustained malignant arrhythmia refractory to conventional antiarrhythmic agents and complicated by heart failure, early ECMO can provide time for myocardial and electrophysiological recovery and is therefore an effective salvage therapy. When genetic testing yields a variant of uncertain significance, the clinical phenotype and treatment response are valuable references for assessing pathogenicity.

## Data Availability

No datasets were generated or analysed during the current study.
